# Supplementary material for: Characteristics, Cryoprotection Evaluation and In Vitro Release of BSA-Loaded Chitosan Nanoparticles
Source: Mar Drugs. 2020 Jun 15;18(6):315. doi: 10.3390/md18060315 (PMC7345782; doi:10.3390/md18060315)
Supplement: Supplementary file 1 [file marinedrugs-18-00315-s001.pdf]

Supplementary materials

Table S1. The Physicochemical Characteristics of CS-NPs prepared by using different concentration of the CS.

C_{CS} (*mg/mL*)	MHD ± SD (nm)	PDI	ζ-pot ± SD (mv)	EE ± SD (%)	LC (%)
1	90±1	0.18	20±1	37±1	59±1
1.5	101±2	0.19	22±1.5	41±2	49±1
2	115±1	0.19	20±0.2	49±0.2	30±0.5
2.5	123±3	0.18	19±1	52±1	24±1
3	128±2	0.16	18±1	66±1	24±1

CS concentration = 1mg/ml.

Results were reported as mean ± SD, n=3.

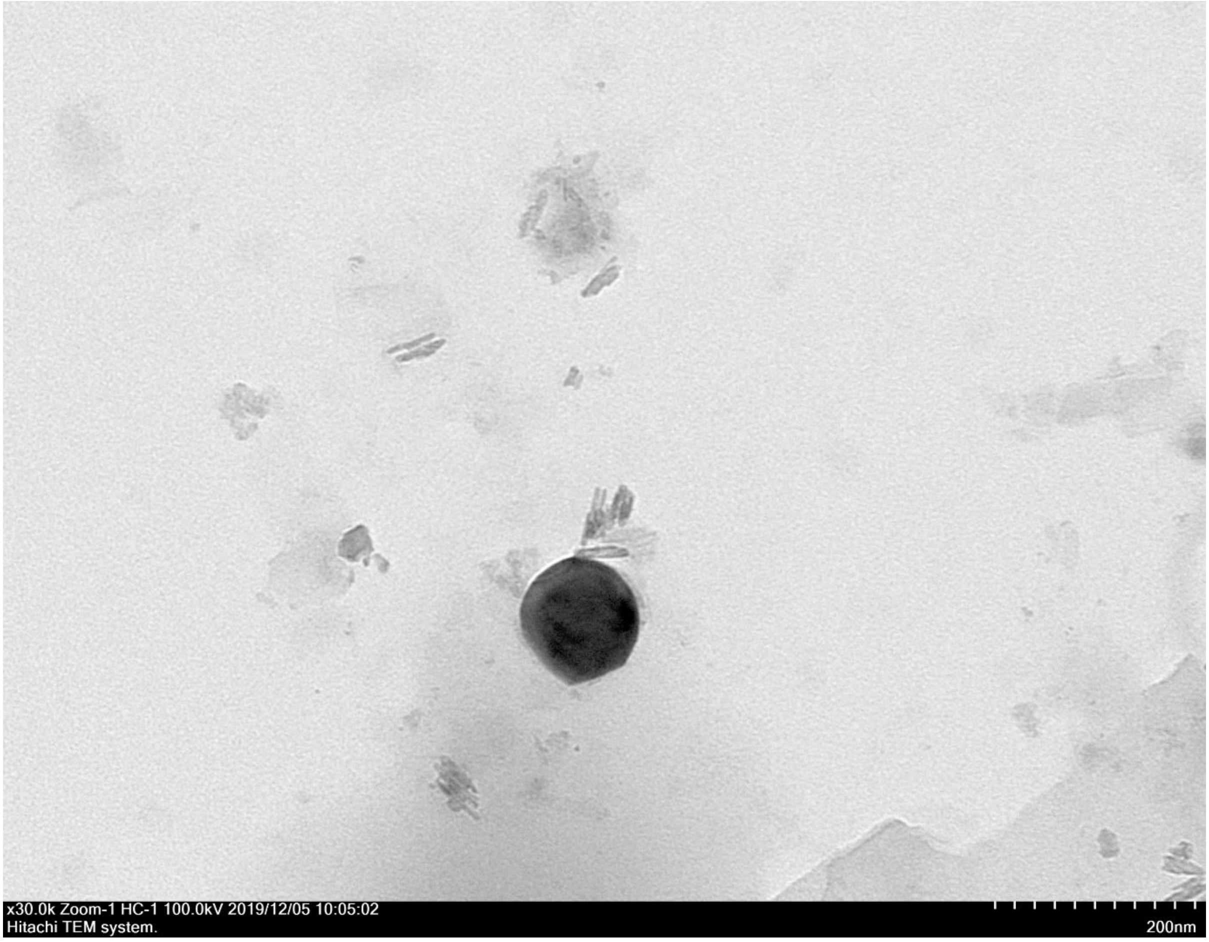

Figure S1. TEM image of BSA-loaded NPs.

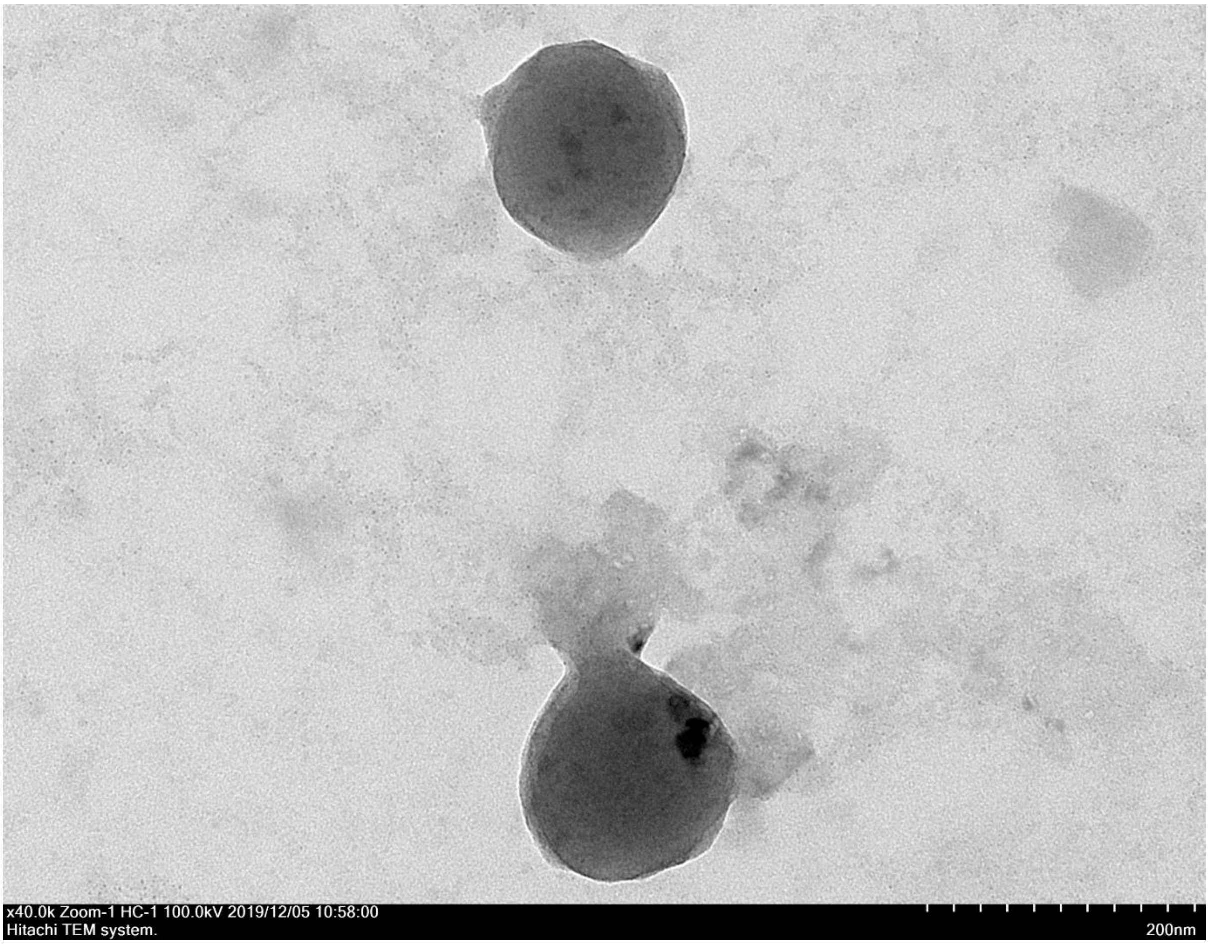

Figure S2. TEM image of re-dissolved BSA-loaded NPs.
